# Effect of Pore Size and Film Thickness on Gold-Coated Nanoporous Anodic Aluminum Oxide Substrates for Surface-Enhanced Raman Scattering Sensor

**DOI:** 10.3390/s151229778

**Published:** 2015-11-30

**Authors:** Aschalew Kassu, Carlton Farley, Anup Sharma, Wonkyu Kim, Junpeng Guo

**Affiliations:** 1 Department of Engineering, Construction Management & Ind. Tech., Alabama A & M University, 4900 Meridian Street, Normal, AL 35762, USA; 2 Department of Physics, Chemistry and Mathematics, Alabama A & M University, 4900 Meridian Street, Normal, AL 35762, USA; farleyc_19@yahoo.com (C.F.); anup.sharma@aamu.edu (A.S.); 3 Department of Electrical and Computer Engineering, University of Alabama in Huntsville, 301 Sparkman Dr., Huntsville, AL 35899, USA; wk0001@uah.edu (W.K.); guoj@uah.edu (J.G.)

**Keywords:** surface enhanced Raman scattering, sensing, nanoporous substrates, anodic aluminum oxide, chemical sensor, optical sensor, ceramic membranes

## Abstract

A sensitive surface enhanced Raman scattering chemical sensor is demonstrated by using inexpensive gold-coated nanoporous anodic aluminum oxide substrates. To optimize the performance of the substrates for sensing by the Surface-enhanced Raman scattering (SERS) technique, the size of the nanopores is varied from 18 nm to 150 nm and the gold film thickness is varied from 30 nm to 120 nm. The sensitivity of gold-coated nanoporous surface enhanced Raman scattering sensor is characterized by detecting low concentrations of Rhodamine 6G laser dye molecules. The morphology of the SERS substrates is characterized by atomic force microscopy. Optical properties of the nanoporous SERS substrates including transmittance, reflectance, and absorbance are also investigated. Relative signal enhancement is plotted for a range of substrate parameters and a detection limit of 10^−6^ M is established.

## 1. Introduction

Surface-enhanced Raman scattering (SERS) spectroscopy is a powerful trace detection technique for chemical sensing. It has been shown that the technique can detect and identify single molecules [1,2]. The SERS technique measures the vibrational frequencies of functional chemical bonds in Raman-active molecules. In order to achieve surface enhanced Raman signals, the shape and the arrangement of metal nanostructures on the SERS substrate play a significant role. Surface enhancement of Raman scattering signals occurs when the excitation light frequency is resonant with the local surface plasmon resonance frequency of the metal nanostructures on the SERS substrates. For SERS-based sensors, most substrates are fabricated by photo-lithography or e-beam lithography. Despite its increasing application and numerous recent SERS investigations reporting an enhancement factor (EF) in the range of 10^12^–10^15^, the technique still suffers from lack of reproducibility and high cost of fabrication of the SERS substrates. To overcome this, in recent years, many studies have been conducted to develop alternate cost effective SERS substrates using wide range of materials, surface morphology, and fabrication techniques. Some of the substrates and the fabrication techniques include gold-coated nanorod arrays fabricated by depositing silicon nanorod arrays onto a silicon and a glass substrate [3], self-assembled gold and silver nanorod arrays [4], nanostructured macroporous silicon substrates [5], arrays of gold nanoparticles and nanotriangles [6], silver-plated porous silicon [7], and ZnO/Au composite nanoarrays [8].

In this work, we use gold coated nanoporous anodic aluminum oxide (NAAO) substrates for SERS chemical sensors. The high density NAAO membranes fabricated using inexpensive non-lithographic method [9,10] are biocompatible [11,12], mechanically robust [9] and can be produced with uniform inter-pore distances [13] in pore sizes ranging between 5–400 nm [14], having a thickness of 100 μm. Due to these benefits, the potential applications of NAAO membranes as an optical, chemical and biosensing platform and devices is widely investigated [15,16,17,18,19]. In our previous work [20], we used a single gold film-thickness of 62 nm on three substrates with pore-size of 35, 80 and 150 nm. A later conference proceeding [21] described preliminary work with four different gold-film thicknesses on a single substrate of 35 nm pore size. The work presented here is substantially more comprehensive where nine different gold-film thicknesses are investigated on substrates with six different pore sizes.

To investigate the effect of gold film thickness on the sensitivity of localized surface plasmon (LSP) enhanced Raman scattering, SERS measurements are made using low concentrations of Rhodamine 6G (Rh6G) dye solution in water and adsorbed on the surface of the gold coated NAAO substrate. The primary motivation for this work is to characterize the novel substrates. Rh6G dye is Raman active molecule widely used for SERS, including single molecule study [2]. Here, the SERS enhancement is attributed to the interaction of LSP with the Rh6G dye deposited on the gold coated NAAO SERS substrates. The plasmonic resonance effect mainly depends on the surface profile of the nanostructure, including the size, shape, and the type of metal used for coating the NAAO SERS substrates [20,22]. More recently, the application of SERS for detection of melamine in milk using gold nanoparticles due to selective binding of melamine with nanoparticles was demonstrated. Signal enhancement for detection of adulterants as low as 0.012 mM is demonstrated [23,24,25]. The rapidity, requirements of minimal to none sample preparation, non-destructivity and sensitivity in detecting samples through transparent containers makes Raman spectroscopy an ideal technique for investigation of food adulterations [25].

## 2. Experimental Section

The NAAO substrates are commercially produced by non-lithographic technique [9]. All the substrates are 10 × 10 mm in dimension, 100 µm thick, and the pore diameters are 18, 35, 55, 80, 100 and 150 nm. For the 18, 35, and 55 nm sizes, the pore densities are 6 × 10^10^, 10^10^, 6 × 10^9^ cm^−2^ respectively, and pore periods are 44, 94, and 143 nm respectively. The pore-density and pore-period values for 80, 100 and 150 nm pore size substrates are 2 × 10^9^ cm^−2^ and 243 nm respectively [9]. It is well documented in literature that, under the same conditions of SERS measurements, silver provides a better enhancement of Raman scattering signal than gold. However, gold is biologically inert. It is chemically and environmentally a more stable plasmonic material than silver [26,27]. In order to make these substrates SERS active, the membranes are coated with gold film thicknesses ranging from 30 nm to 120 nm with a sputtering system, Denton Discovery 18. Base pressure of the chamber is 5 × 10^−^^6^ Torr. Deposition rate is 0.389 nm per second with 5 × 10^−^^3^ Torr argon pressure and 200 Watt DC power. We have seen that the substrates coated with gold layer remain unaffected for several months. While silver is known to provide a slight improvement in SERS efficiency, gold-coated substrates were used in this work for the following reasons: (1) the surface plasmon resonance for silver is generally at shorter wavelengths compared to gold [27,28,29] and so it will be less suitable for the 785 nm laser used in this work; (2) silver films oxidize relatively easier than gold and so has a more limited lifetime [26,27].

The chemical used to demonstrate SERS is low concentration solution of Rh6G dye (Eastman Kodak) purchased in powder form. The typical concentration of the Rh6G dye used for SERS study is 8 × 10^−5^ M in water-based solution. This is the lowest concentration which was detected using the different nanopore substrates coated with gold film thicknesses ranging from 30 nm to 120 nm. A small droplet (20 μL) of diluted Rh6G dye solution was pipetted onto gold coated NAAO SERS substrates and allowed to air-dry. Results for a signal integration time of 3 s are reported here.

USB 2000 UV-VIS-ES spectrometer integrated with DH-2000 UV-VIS-NIR Deuterium-Halogen lamp, (Ocean Optics, Dunedin, FL, USA) are used to study the reflectance and transmittance of the NAAO substrates before and after gold coating. The SERS measurements are collected using 785 nm laser from Enwave Optronics Raman system. The details of the experimental setup for optical characterization and SERS measurements are described elsewhere [20,21]. The SERS substrates with the adsorbed chemical were placed at an optimum distance of 7 mm from the probe. The SERS signals from Rh6G dye on the gold-coated NAAO are integrated over time periods ranging between 1 s to 2 min. However, the SERS measurements presented in this paper are recorded for integration time of 3 s.

## 3. Results and Discussion

This study provides a methodology to systematically modify the resonances of gold coated nanoporous SERS substrates by changing the pore sizes and gold film thickness to enhance the intensity of Raman signals. We investigated SERS on NAAO substrates with pore diameters of 18, 35, 55, 80, 100 and 150 nm coated with 30, 40, 50, 60, 70, 80, 90, 100, and 120 nm thick gold film. The gold coated nanopores are much smaller in size than the laser wavelength. This is known to enhance the momentum of the surface plasmons by scattering the light from the pores coated with plasmonic metal films. This, in turn, provides local-field enhancement and the enhancement of the Raman scattering signal from the Rh6G molecule absorbed on the surface of the SERS substrate [26,30]. The transmittance and reflectance of all substrates were measured before and after gold coating. To optimize the substrates and characterize the sensing technique, Raman scattered light signal from Rh6G dye deposited on gold-coated NAAO substrates from a dilute solution (8 × 10^−5^ M) in water was measured.

**Figure 1:**
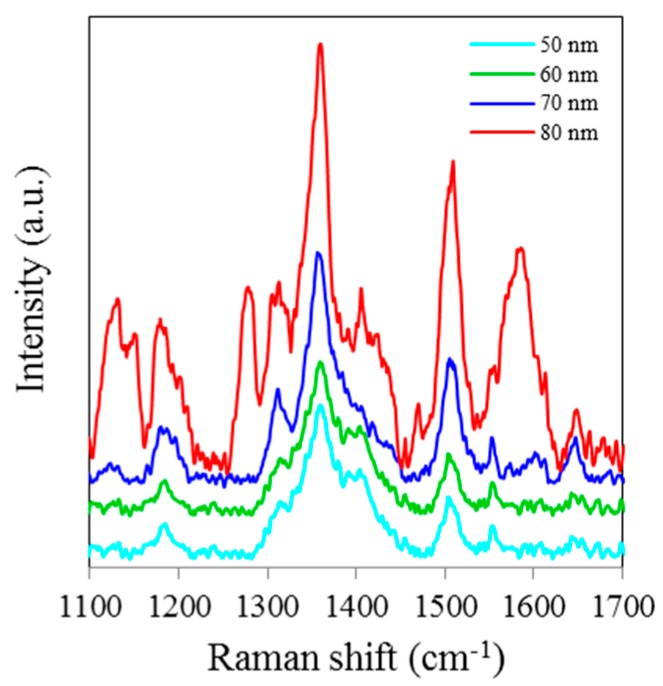
SERS spectra of 8 × 10^−5^ M solution of Rh6G dye adsorbed on the surface of 18 nm NAAO substrate coated with 50, 60, 70 and 80 nm gold films measured with a single scan averaging and an integration time of 3 s.

Figure 1 shows SERS spectra of Rh6G dye adsorbed on the surface of 18 nm NAAO substrate, which is coated with 50, 60, 70 and 80 nm thick gold films. The integration time for these measurements is 3 s. As can be seen, Rh6G Raman spectrum from NAAO substrate coated with 70 nm thick gold film was relatively the most intense. With a 50 nm thick gold film, the characteristic Raman band for Rh6G dye at 1646 cm^−1^ is barely detected. As the thickness of the deposited gold film is increased to 70 nm, the Raman bands, including the weak signal at 766 (not shown) and 1646 cm^−1^ appear to be increasingly intense. As the film thickness is increased beyond 70 nm, the intensity of the Raman peaks starts to diminish. This indicates that for a NAAO substrate with 18 nm pore diameter, the gold film thickness for optimal resonant enhancement of Raman molecular signatures is about 70 nm.

**Figure 2:**
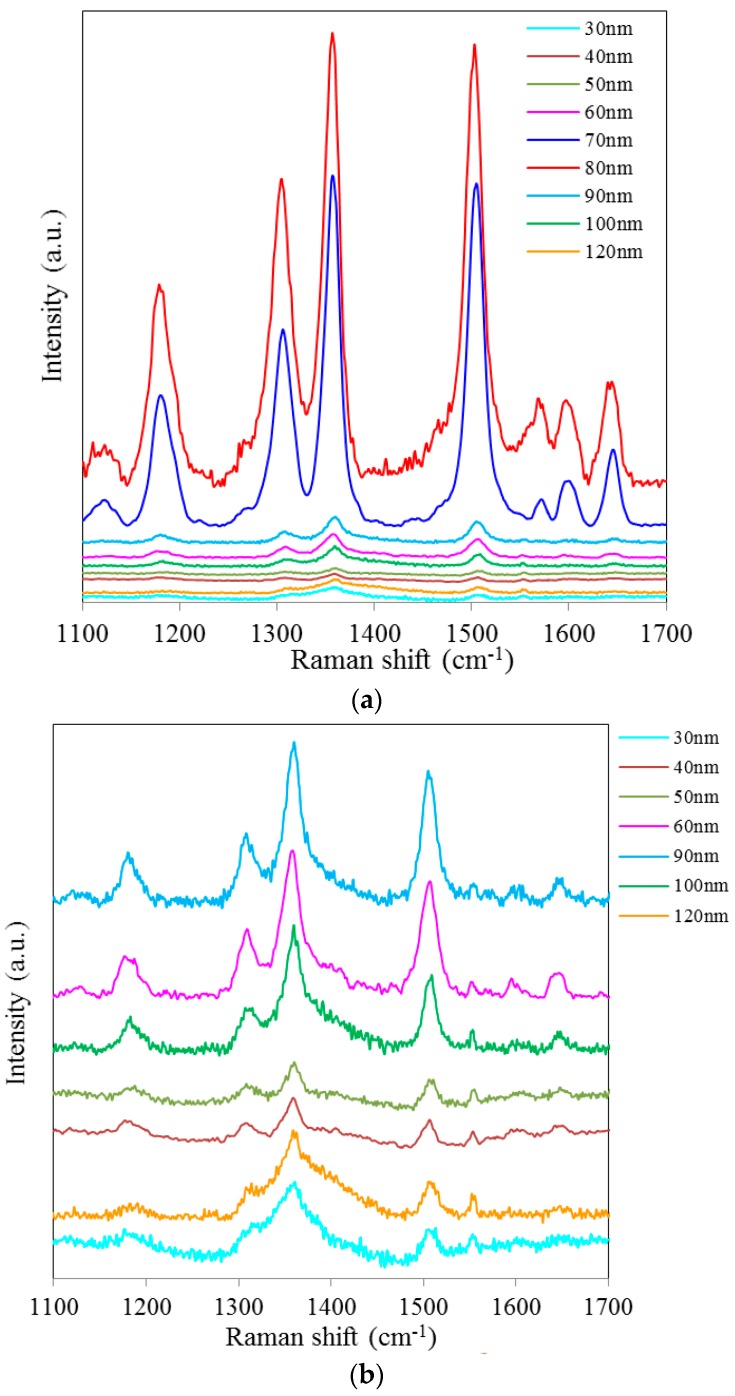
SERS spectra of 8 × 10^−5^ M solution of Rh6G dye adsorbed on the surface of 35 nm NAAO substrate coated with 30, 40, 50, 60, 70, 80, 90, 100 and120 nm gold films measured with 3 s integration time (**a**); Figure (**b**) shows the relative enhancement of the weak Raman signals after removing the spectra for the 70 and 80 nm gold coated 35 nm pore size substrate measured with 3 s integration time.

**Figure 3:**
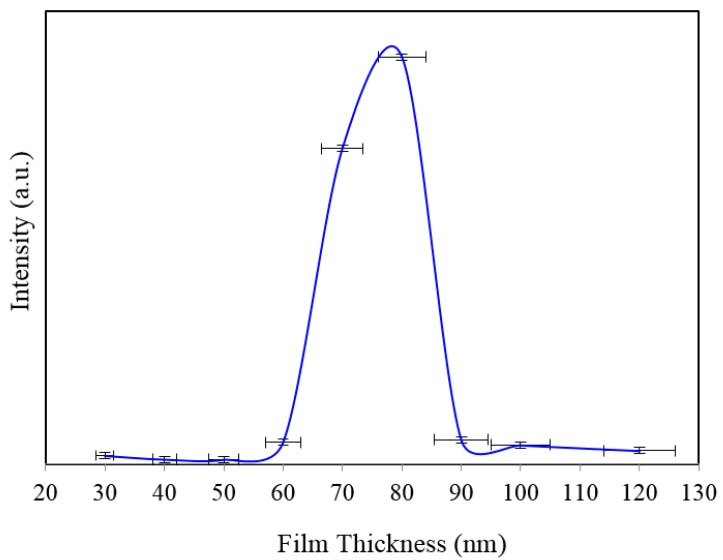
Peak SERS intensities measured at about 1361 cm^−1^ measured from 8 × 10^−5^ M solution of Rh6G dye adsorbed on the surface of 30, 40, 50, 60, 70, 80, 90, 100 and 120 nm gold films deposited on 35 nm nanoporous SERS substrate with 3 s integration time.

Figure 2a shows SERS spectra of Rh6G dye adsorbed on the surface of 35 nm NAAO substrate coated with 30, 40, 50, 60, 70, 80, 90, 100 and 120 nm gold films measured with an integration time of 3 s. For this substrate, the strongest SERS signals were obtained for a gold coating of 80 nm. As can be seen in this figure, the intensity of SERS signals falls significantly as the thickness of gold film is increased to 90 nm or decreased to 60 nm. For 70 and 80 nm gold films, the Raman bands at 606, 767, 1180, 1306, 1360, 1506, and 1630 cm^−1^ are clearly visible. For other film thicknesses, only the prominent Raman bands at 1361 and 1506 cm^−1^ are seen (Figure 2b). As the gold film thickness approaches 60 nm, the Raman peaks at 606, 767, 1180 and 1600 cm^−1^ start to appear and get enhanced until the film thickness equals 80 nm and starts fading after that. In order to see the relative enhancement of the weak Rh6G Raman signatures from the 35 nm size porous substrate coated with 30, 40, 50, 60, 90, 100 and 120 nm gold thickness, the spectra for 70 nm and 80 nm thick gold coated SERS substrates (shown in Figure 2a) are removed and the rest are shown in Figure 2b. Here, we can clearly see that the SERS signal from the 60, 90 and 100 nm thick gold coated substrate show a relatively better enhancement than the 30, 40, 50 and 120 nm thick gold coated 35 nm size porous substrates. Comparison of peak SERS intensity for 1361 cm^−1^ band of Rh6G dye adsorbed on the surface of 30, 40, 50, 60, 70, 80, 90, 100 and 120 nm thick gold films deposited on 35 nm NAAO SERS substrate at 3 s integration time is shown in Figure 3. As can be seen in the figure, for the 35 nm hole sizes, the resonance gold film thickness required to design a relatively high performance SERS substrate is about 80 nm. Large thickness of gold-film has the potential to cover the nanopore and reduce SERS enhancement. Thus, it is interesting that the optimum gold-film thickness (80 nm) is much larger than the pore-size (35 nm). This can be explained by the fact that nanopores in these substrates are hollow nano-cylinders and the actual gold-film thickness on the vertical walls of these nanopores is much less than the measured thickness on horizontal surface of the substrate.

**Figure 4:**
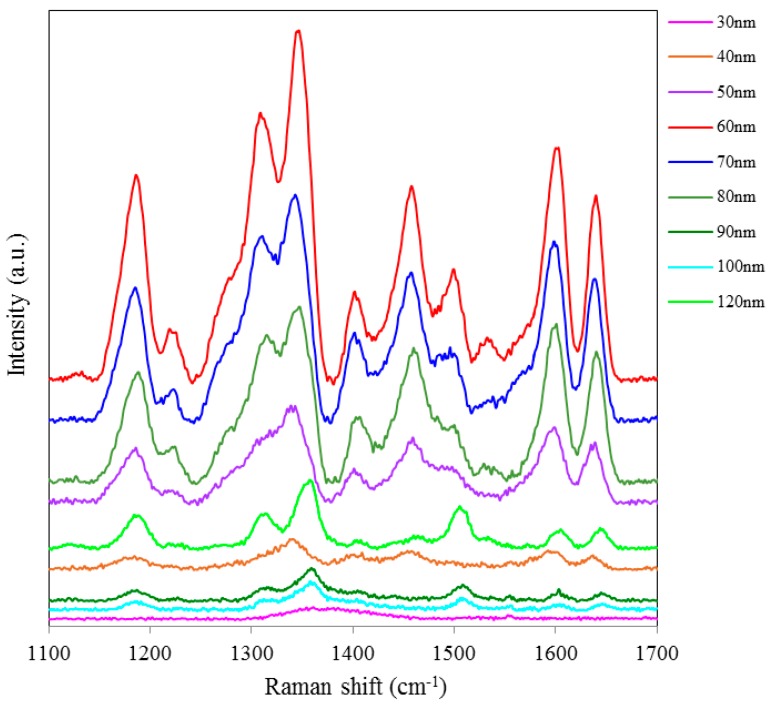
SERS spectra of 8 × 10^−5^ M solution of Rh6G dye adsorbed on the surfaces of 55 nm nanoporous substrate coated with 30, 40, 50, 60, 70, 80, 90, 100 and 120 nm gold films, measured with 3 s integration time.

**Figure 5:**
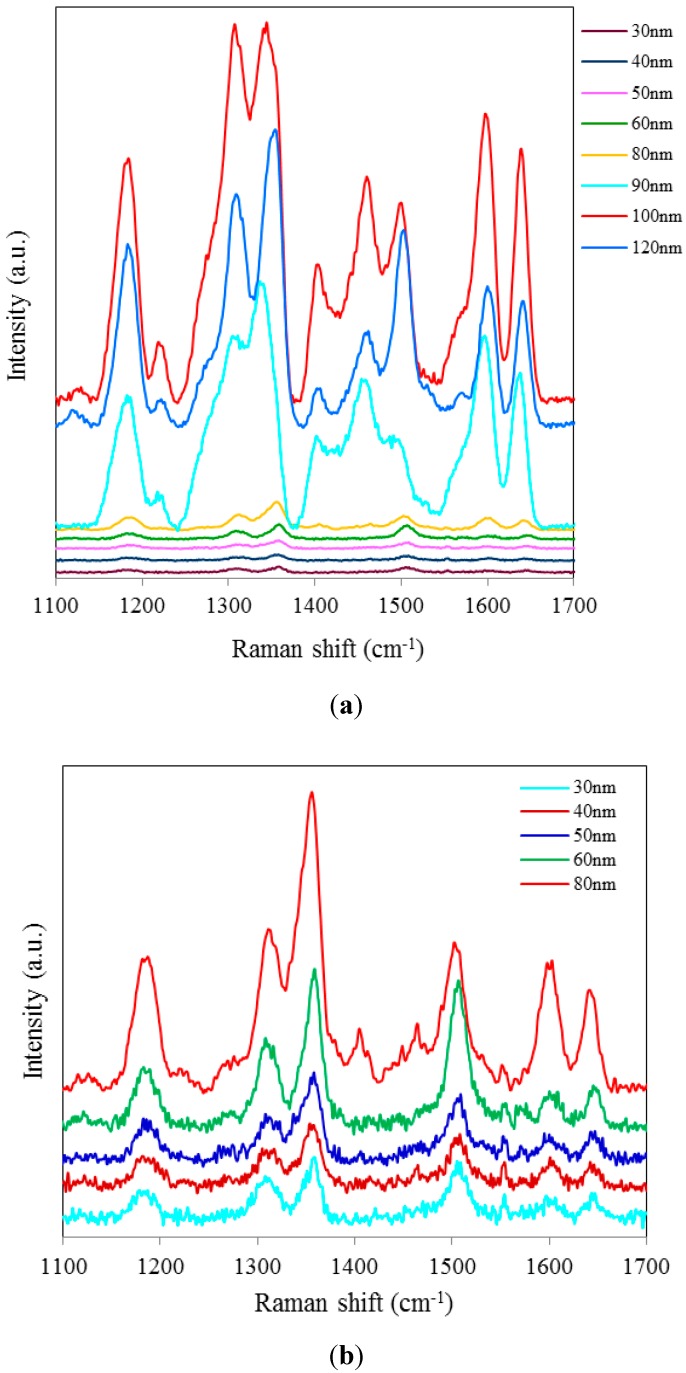
SERS spectra of 8 × 10^−5^ M solution of Rh6G dye adsorbed on the surface of 80 nm NAAO nanoporous substrate coated with 30, 40, 50, 60, 70, 80, 90, 100, 120 nm gold films measured with 3 s integration time (**a**); Figure (**b**) shows the relative enhancement of the weak Raman signals after removing the spectra for the 90, 100, and 120 nm gold coated 80 nm pore size substrate measured with 3 s integration time.

**Figure 6:**
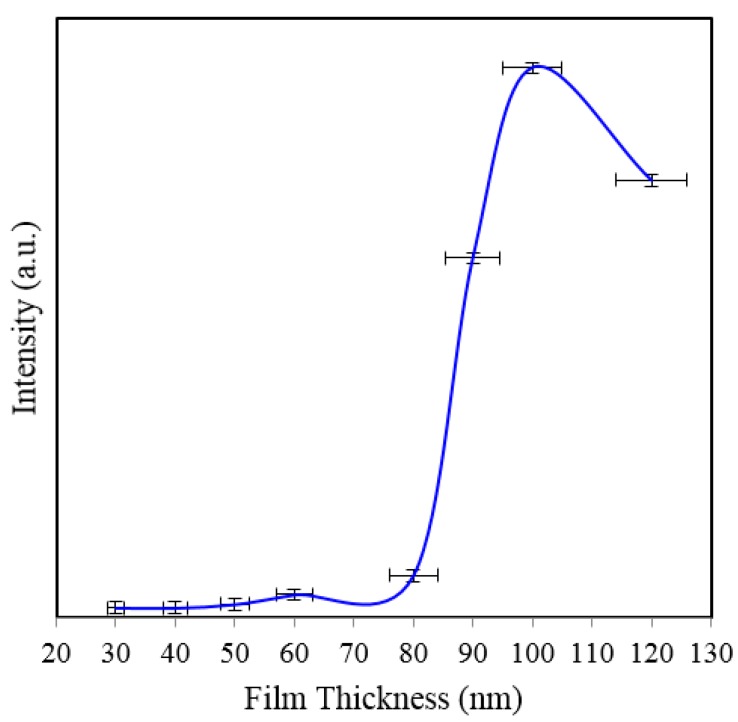
Peak SERS intensities measured at about 1361 cm^−1^ measured from 8 × 10^−5^ M solution of Rh6G dye adsorbed on the surface of 30, 40, 50, 60, 80, 90, 100 and 120 nm gold films deposited on 80 nm NAAO nanoporous substrate with 3 s integration time.

**Figure 7:**
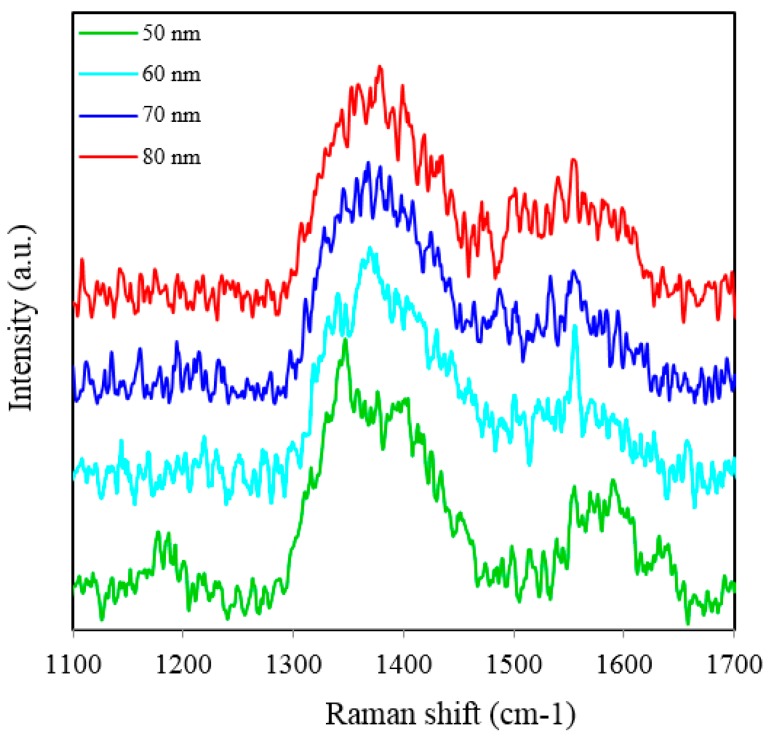
SERS spectra of 8 × 10^−5^ M solution of Rh6G dye adsorbed on the surface of 150 nm nanoporous substrate coated with 50, 60, 70 and 80 nm gold films measured with 3 s integration time.

SERS spectra of Rh6G dye adsorbed on the surface of 55 nm NAAO substrate coated with 30, 40, 50, 60, 70, 80, 90, 100 and 120 nm gold films measured at 3 s integration time is shown in Figure 4. For this substrate, the optimum gold-film thickness is 60 nm. As compared with the SERS measurements made with the 55 nm SERS substrates, the characteristic Raman bands measured from 35 nm pore SERS substrates are narrower and more prominent. Likewise, Figure 5 shows SERS spectra of Rh6G dye adsorbed on the surface of 80 nm NAAO substrate coated with 30, 40, 50, 60, 70, 80, 90, 100, and 120 nm gold films and measured at 3 s integration time. The Raman measurements from the 80 nm pore size substrates resulted in intense Raman bands (Figure 5a) for relatively thicker gold films (90, 100 and 120 nm), as compared with the measurements made with the SERS substrates with pore-sizes of 35 nm and 55 nm, coated with the 90–120 nm gold thickness. Here, to compare the relative enhancement of the weak Rh6G Raman signatures from the 80 nm size porous substrate coated with 30, 40, 50, 60 and 80 nm gold thickness, the spectra for 90, 100 and 120 nm thick gold coated SERS substrates shown in Figure 5a are removed and the rest are shown in Figure 5b. We can clearly see that the SERS signal from the 80 nm gold coated 80 nm pore size substrate shows a relatively better enhancement of the Raman bands followed by 60, 50, 40, 30 nm coated SERS. Peak SERS intensity for 1361 cm^−1^ band of Rh6G on 30, 40, 50, 60, 80, 90, 100 and 120 nm gold films deposited on 80 nm NAAO SERS substrate at 3 s integration time is shown in Figure 6. As explained earlier, the actual gold film-thickness on the vertical walls of the hollow nano-cylinders is much less than the measured values and this explains the low SERS enhancement for thicknesses up to 80 nm. The horizontal error-bar shows an uncertainty of 5% in the film thickness as quoted for the sputtering device used. The SERS measurements collected from the 90, 100 and 120 nm thick gold coated 80 nm pore size SERS substrates shows a slight shift of 1361 cm^−1^ band which is within the line-width. We believe this apparent shift is a result of the variation of relative intensity of two adjacent bands. Such an effect has been observed by others also [31]. SERS spectra of Rh6G dye on the surface of 150 nm NAAO substrate coated with 50, 60, 70 and 80 nm gold films measured at 3 s integration time is shown in Figure 7. Interestingly, in this case, the overall enhancement of SERS is insignificant and no considerable variation of Raman signal from the substrate deposited with 50 to 80 nm gold films is observed. This is because of the fact that the local field enhancement and the relative SERS signal enhancement largely depend on the geometric parameters of the substrate, including nanopore size and thickness of the metallic film. The increase in the inter-particle distance can lead to a decrease of electromagnetic field enhancement near the surface of the gold coated nanopore SERS substrate [20,30]. Due to this, for the SERS measurements collected from the larger pore size SERS substrates (Figure 7), the signal-to-noise ratio of the spectra and the relative SERS signal enhancement are low as compared with the SERS measurements collected from smaller pore size substrates [30] shown in Figure 2 and Figure 4.

**Figure 8:**
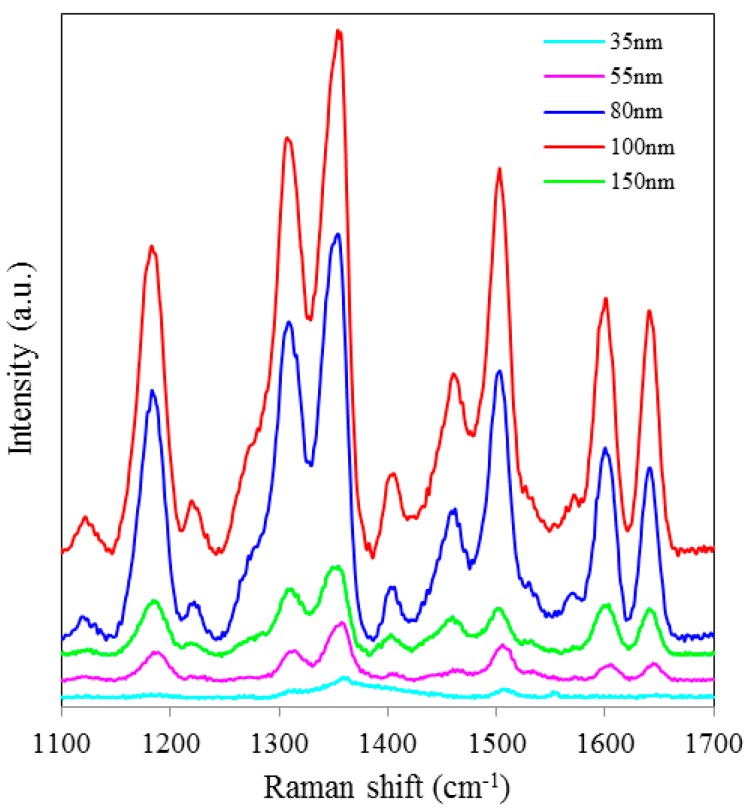
SERS spectra of 8 × 10^−5^ M solution of Rh6G dye adsorbed on the surface of 120 nm thick gold film deposited on 35, 55, 80, 100, and 150 nm nanoporous substrates measured at 3 s integration time.

**Figure 9:**
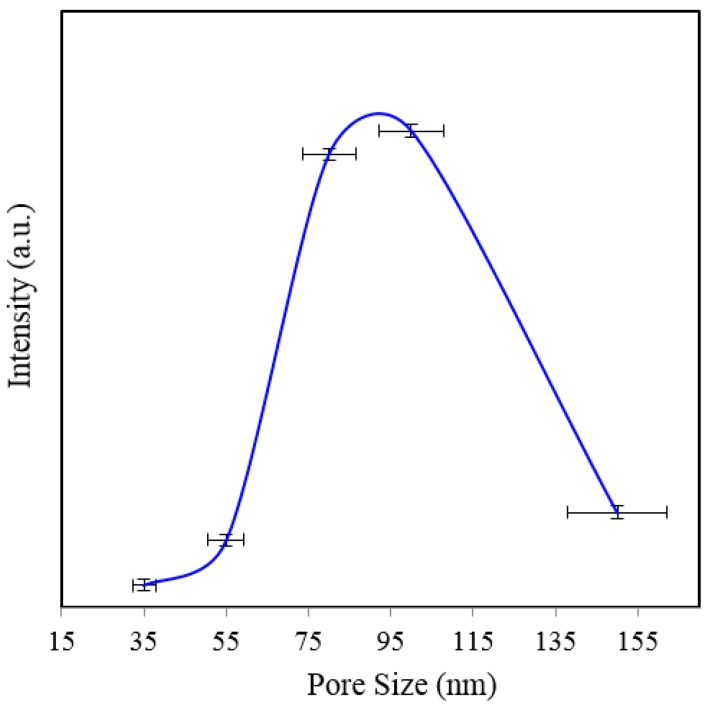
Comparison of SERS intensities at about 1361 cm^−1^ measured from 8 × 10^−5^ M solution of Rh6G dye adsorbed on the surface of 120 nm gold films deposited on 35, 55, 80, 100, and 150 nm NAAO SERS substrate at 3 s integration time.

**Figure 10:**
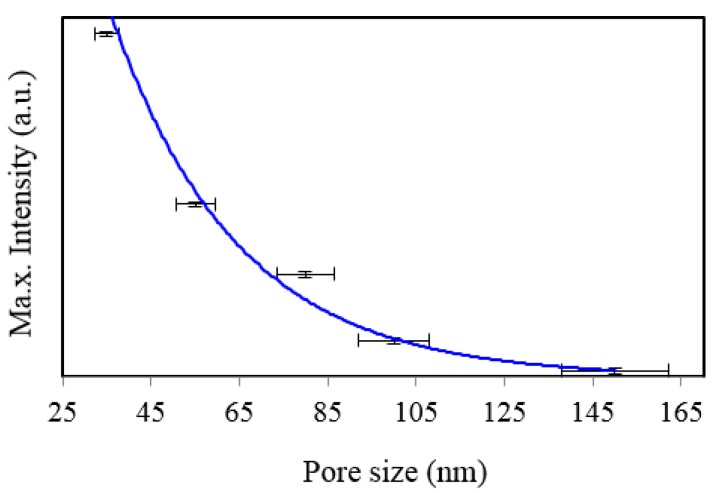
Peak SERS intensities measured at 1361 cm^−1^ measured from 8 × 10^−5^ M solution of Rh6G dye adsorbed on the surface of 50–80 nm thick gold films deposited on 35, 55, 80, 100, and 150 nm pore size substrate at 3 s integration time.

To compare the effect of thicker gold film on the SERS signal, 120 nm thick gold films are sputter coated on 35, 55, 80, 100 and 150 nm NAAO substrates. The SERS spectra of Rh6G dye adsorbed on these substrates is measured at 3 s integration time and shown in Figure 8. Here, we can see that the 100 and 80 nm pore size NAAO substrates resulted in a relatively significant Raman enhancement followed by 150, 55 and 35 nm pore size substrates respectively. The SERS enhancement measured from the smaller pore sizes (35, and 55 nm) and the largest pore size substrates (150 nm) is relatively insignificant as compared with the 120 nm thick gold coated 80 and 100 nm NAAO substrates. However, as can be seen in Figure 8 and Figure 9, for the 120 nm gold deposited films, the SERS enhancement of the largest pore size (150 nm) are prominent as compared with 35 nm pore substrate. This effect can be explained by the fact that the 120 nm gold film deposited on the 35 nm pore size SERS substrates can fill the pores making the surface relatively smooth, which reduces the SERS efficiency. Comparison of SERS intensities for 1361 cm^−1^ Raman band of Rh6G on the surface of 120 nm gold films deposited on 35, 55, 80, 100, and 150 nm NAAO SERS substrate at 3 s integration time is shown in Figure 9. The results showing relative performance of substrates coated with 50–80 nm thick gold films measured by the intensity of 1361 cm^−1^ band are shown in Table 1 and Figure 10. As seen in Figure 10, the peak intensity of 1361 cm^−1^ is the smallest for 150 nm pore-size substrate (Figure 7). While the noise amplitude is about the same for this substrate, the signal-to-noise ratio is poor as expected.

**Table 1:** Relative performance of the 50–80 nm thickness gold coated substrates measured by the intensity of 1361 cm^−1^.

Film Thickness (nm)	Pore Sizes (nm)				
	35 nm	55 nm	80 nm	100 nm	150 nm
50	85	373	128	225	112
60	408	1568	2210	282	127
70	5919	3842	2280	805	133
80	7633	1720	1780	203	137

**Figure 11:**
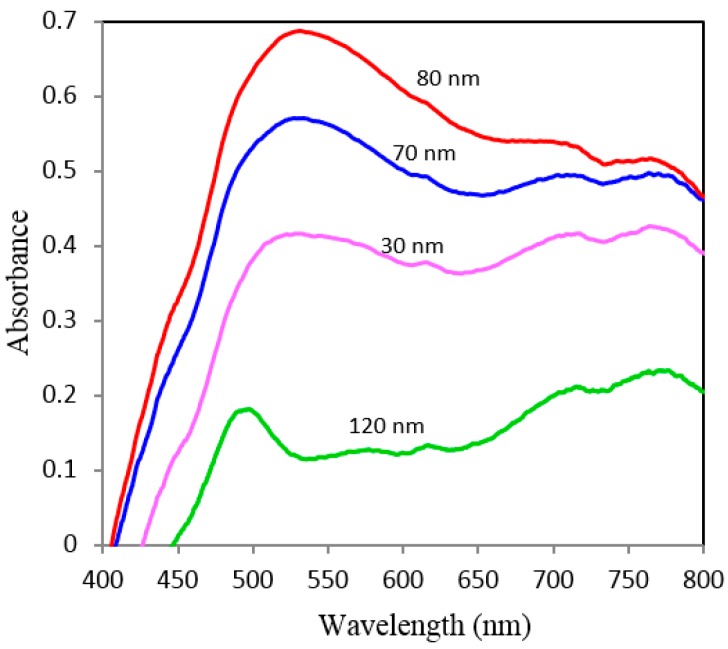
Normalized absorption spectra for 35 nm pore size coated with 70, 80, 90 and 120 nm thick gold films measured at normal incidence.

**Figure 12:**
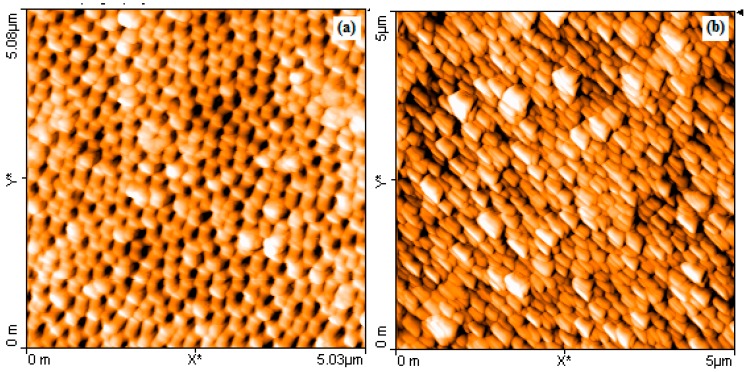
AFM micrographs of 80 nm pore size NAAO substrate before (**a**); and after depositing 70 nm thick gold film using the sputtering technique (**b**).

Representative absorption spectra and Atomic Force Microscopy (AFM) micrographs of NAAO SERS substrates are shown in Figure 11 and Figure 12 respectively. The horizontal error-bars in Figure 9 and Figure 10 show the size-variation of nanopores as can also be seen in the AFM micrographs of Figure 12. The broad absorption spectra (Figure 11), shows the role of LSP effect in response to gold coated nanoporous SERS substrate [26]. The LSP spectrum of gold-coated substrates has a very broad maximum with little variation between 550 nm and 785 nm. Others have used 785 nm laser on substrates with much narrower resonance between 500–600 nm [22,26]. Our choice was also decided by the availability of this light source.

It is also observed that for each nanopore size, there is a clear variation in the intensity of Raman spectra with change in the thickness of gold film. SERS enhancement and limit of detection of the substrates largely depend on the pore size, the optimum gold thickness for that particular pore size [32,33], and the surface roughness and morphology of the SERS substrates [33,34]. It is well understood that efficiency of gold-coated SERS substrates depends not only on the substrate roughness but also on the thickness of the coated gold-film [33,34]. Li *et al.* [33] studied the effects of gold film morphology on surface plasmon and SERS using periodic nanostructured P3HT:PMMA/Au {poly(3-hexyl thiophene) and poly (methyl methacrylate)} on silicon substrates with a wide range of hole size and lattice constants. It is shown that, as the thickness of the nanopores increases and the pore size decreases, there will be a coupling effect between the neighboring pores leading to enhancement of resonance localized electric field. The study concluded that SERS enhancement is largely dominated by the structures of the pore array than the roughness of the SERS substrate. In another work [34], the effects of the thickness of a plasmonic metal films and the surface roughness on SERS substrate fabricated on glass polydimethylsiloxane (PDMS) templates was studied, and it was shown that the role of the thickness of the metallic film in SERS enhancement is more significant than the surface roughness. Since NAAO substrates, with such a wide range of pore sizes and gold film thickness have only been investigated by us, we believe it is appropriate to characterize SERS efficiency based on the film-thickness and pore size. While, on one hand, one needs a certain minimum thickness of gold-film for SERS to work, by over-coating the AAO substrates one will fill the pores and reduce the SERS effect as well as the limit. As is borne out by our results, there is an optimum thickness for maximum SERS efficiency. For most of the pore-sizes investigated here, the optimum film-thickness is in the range of 70–80 nm (Table 1).

It is well known [35] that (unlike regular Raman) while SERS is widely used to sense chemicals at very small concentrations, it has limited utility for quantitative analysis. This is especially so for a non-periodic substrate like the one investigated here. It is known that a slight change in surface morphology, as in non-periodic substrates, can result in dramatic changes in signal enhancement and reproducibility for a quantitative measurement is questionable. The objective of this work is to optimize substrate parameters like pore-size and gold-film thickness for maximum relative signal-enhancement. In general, for all NAAO pores size SERS substrates, the SERS intensity increases as the film thickness increases from 30 nm until it reaches the optimum thickness corresponding to a specific pore size and increasing the film thickness above the optimum value results in a gradual decrease in the intensity of Raman signal.

Several SERS measurements were made at different locations of the Rh6G spotted area on the SERS substrates. The intensity of the Raman signal depends on the spot where the measurement is collected. The measurements collected from the SERS “hot-spot” results in large enhancement of Raman signal as compared with the other spots. Due to the intensity variation of the SERS measurement resulting from the irregular “hot-spot” locations throughout the SERS substrate [30], it is impossible to perform linearity analysis for quantitative chemical sensing. Based on multiple measurements on a given substrate we have added error-bars on Figure 3, Figure 6, Figure 9 and Figure 10. From the signal-to-noise ratio of measured spectra a limit of detection of 10^−6^ M can be established. For the 35, 80 and 150 nm pore size substrates coated with about 62 nm gold films, the calculated value of the EFs are about 10^5^, 8.8 × 10^4^ and 8.2 × 10^4^ respectively [20] and the detection limit for 35 nm pore coated with about 62 nm thick gold film is about 10^−6^ M Rh6G [20]. EF is used as a general measure of performance of SERS substrate estimated through approximations of several measurement parameters [36]. Hence, a direct comparison of the SERS EFs of the substrates used in this study with literature values obtained with different SERS substrates, fabrication techniques and other parameters is not suggested [36]. While other substrates with much higher EFs are known, we believe the NAAO substrates reported here can have applications where inexpensive substrates with relatively lower EFs are required.

## 4. Conclusions

Effect of gold film thickness and nanopore size on SERS signal is investigated by using low cost aluminum oxide nanoporous substrates of 18, 35, 55, 80, 100 and 150 nm porous sizes. The optimum film thickness for the resonance condition depends on the nanoporous size of the NAAO substrates. In general, the 18, 35, and 55 nm size SERS substrates with 50 to 80 nm thick gold film resulted in narrow and large enhancement of most of the characteristics Rh6G Raman bands at 606, 767, 1180, 1306, 1360, 1506, and 1630 cm^−1^. The nanopores deposited with thin gold film (30 and 40 nm) and relatively thick films (90, 100, and 120 nm) all resulted in a relatively insignificant enhancement in Raman signal. For the 80 nm pore size substrates, the Raman measurements collected from the substrates deposited with relatively thicker gold films (90 to 120 nm) resulted in intense Raman bands as compared with the measurements collected from the substrates deposited with gold film thickness ranging from 30 to 80 nm. However, the overall enhancement of Raman signals from the substrates coated with gold film thicknesses in the range of 50 to 80 nm is insignificant. As mentioned in the earlier section, these results can be explained, as due to the vertical wall geometry of the hollow nano-cylinders, whereby the actual thickness of gold-film is much less than the measured thickness on horizontal surface of the substrate. The results clearly demonstrate the utility of these inexpensive gold-coated nanoporous aluminum substrates for applications involving SERS. Due to the random position and non-periodic nature of these nanopores (Figure 12), the experimental results are not easy to reproduce by theoretical modelling. Our effort for modelling using a commercial Lumerical finite-difference time-domain (FDTD) Solution technique to understand the effect of pore size and gold film thickness on absorption due to surface plasmon produced spectra did not agree with the results in Figure 11. Due to unreliability of the modelling technique, these results are not shown.
